# CFTR Correctors and Antioxidants Partially Normalize Lipid Imbalance but not Abnormal Basal Inflammatory Cytokine Profile in CF Bronchial Epithelial Cells

**DOI:** 10.3389/fphys.2021.619442

**Published:** 2021-02-04

**Authors:** Mieke Veltman, Juan B. De Sanctis, Marta Stolarczyk, Nikolai Klymiuk, Andrea Bähr, Rutger W. Brouwer, Edwin Oole, Juhi Shah, Tomas Ozdian, Jie Liao, Carolina Martini, Danuta Radzioch, John W. Hanrahan, Bob J. Scholte

**Affiliations:** ^1^Cell Biology Department, Erasmus Medical Center, Rotterdam, Netherlands; ^2^Pediatric Pulmonology, Sophia Children’s Hospital, Erasmus Medical Center, Rotterdam, Netherlands; ^3^Faculty of Medicine and Dentistry, Institute of Molecular and Translational Medicine, Palacký University, Olomouc, Czechia; ^4^Large Animal Models for Cardiovascular Research, TU Munich, Munich, Germany; ^5^Center for Innovative Medical Models, LMU Munich, Munich, Germany; ^6^Center for Biomics, Erasmus Medical Center, Rotterdam, Netherlands; ^7^Department of Medicine, The Research Institute of the McGill University Health Centre, McGill University, Montreal, QC, Canada; ^8^Department of Physiology, CF Translational Research Centre, McGill University, Montreal, QC, Canada

**Keywords:** cytokine array, cystic fibrosis transmembrane conductance regulator corrector therapy, oxidative stress, polyunsaturated (essential) fatty acids, ceramide species, bronchial epithelial cell, lipidomics, cystic fibrosis

## Abstract

A deficiency in cystic fibrosis transmembrane conductance regulator (CFTR) function in CF leads to chronic lung disease. CF is associated with abnormalities in fatty acids, ceramides, and cholesterol, their relationship with CF lung pathology is not completely understood. Therefore, we examined the impact of CFTR deficiency on lipid metabolism and pro-inflammatory signaling in airway epithelium using mass spectrometric, protein array. We observed a striking imbalance in fatty acid and ceramide metabolism, associated with chronic oxidative stress under basal conditions in CF mouse lung and well-differentiated bronchial epithelial cell cultures of CFTR knock out pig and CF patients. Cell-autonomous features of all three CF models included high ratios of ω-6- to ω-3-polyunsaturated fatty acids and of long- to very long-chain ceramide species (LCC/VLCC), reduced levels of total ceramides and ceramide precursors. In addition to the retinoic acid analog fenretinide, the anti-oxidants glutathione (GSH) and deferoxamine partially corrected the lipid profile indicating that oxidative stress may promote the lipid abnormalities. CFTR-targeted modulators reduced the lipid imbalance and oxidative stress, confirming the CFTR dependence of lipid ratios. However, despite functional correction of CF cells up to 60% of non-CF in Ussing chamber experiments, a 72-h triple compound treatment (elexacaftor/tezacaftor/ivacaftor surrogate) did not completely normalize lipid imbalance or oxidative stress.

Protein array analysis revealed differential expression and shedding of cytokines and growth factors from CF epithelial cells compared to non-CF cells, consistent with sterile inflammation and tissue remodeling under basal conditions, including enhanced secretion of the neutrophil activator CXCL5, and the T-cell activator CCL17. However, treatment with antioxidants or CFTR modulators that mimic the approved combination therapies, ivacaftor/lumacaftor and ivacaftor/tezacaftor/elexacaftor, did not effectively suppress the inflammatory phenotype.

We propose that CFTR deficiency causes oxidative stress in CF airway epithelium, affecting multiple bioactive lipid metabolic pathways, which likely play a role in CF lung disease progression. A combination of anti-oxidant, anti-inflammatory and CFTR targeted therapeutics may be required for full correction of the CF phenotype.

## Introduction

Abnormal lipid levels in blood and lungs are a hallmark of cystic fibrosis (CF). Previous studies of CF children have demonstrated abnormal levels of polyunsaturated fatty acids (PUFA), sphingolipids, and several lipid markers of oxidative stress, and these defects correlate with CF inflammatory lung disease and tissue remodeling in patients (Scholte et al., 2019; Horati et al., 2020) and in animal models (Freedman et al., 1999; Guilbault et al., 2009; Veltman et al., 2016). In particular, these studies revealed higher levels of the oxidative stress markers methionine sulfoxide (Chandler et al., 2018), isoprostanes, and lysolipids (Scholte et al., 2019; Horati et al., 2020) in broncho-alveolar lavage fluid (BALF) from human CF infants compared to non-CF infants, despite comparable inflammation and a lack of detectable bacterial infection. Furthermore, we reported an increased ratio of long-chain to very long-chain ceramide species (LCC/VLCC; Scholte et al., 2019), confirming results with plasma from CF patients and in lung tissue and plasma from CF transmembrane conductance regulator (CFTR) knockout mice (Garic et al., 2017). Moreover, the increase in LCC/VLCC ratio in BALF from CF infants was positively correlated with chest computed tomography (CT) scores and multiple pro-inflammatory markers (Scholte et al., 2019; Horati et al., 2020) and altered recruitment, differentiation, and activity of neutrophils in the BALF of CF infants (Margaroli et al., 2019). Using the Cftr^tm1eur^ F508del CFTR mouse model, in the absence of bacterial infection, we observed abnormal sphingosine phosphate (S1P) metabolism that correlated with enhanced lung macrophage and neutrophil infiltration, abnormal polarization of dendritic cells, increased mucus production, and enhanced responses to Lipo-saccharide (LPS) challenge, which could be partially corrected with a S1P lyase inhibitor (Veltman et al., 2016). The extent to which changes in lipid metabolism contribute to inflammation and tissue remodeling in CF lung disease remains to be established. Hyperinflammation in CF lungs has been extensively studied (Murphy and Ribeiro, 2019). In F508del CFTR mice abnormal basal lipid metabolism coincides with enhanced responses to the pro-inflammatory and pro-fibrotic triggers LPS (Merkert et al., 2019), bleomycin (Huaux et al., 2013), and *Pseudomonas* infection (Palomo et al., 2014). Whereas lysolipids and S1P are known to activate receptors and influence lung pathology, a change in lipid content, in particular, ceramide species and PUFA ω6–ω3 ratio is more likely to affect membrane microdomains, membrane trafficking, and the activity of membrane-associated transport and receptor systems that control basal and induced inflammation and tissue repair. In support of this, arachidonic acid/docosahexaenoic acid (AA/DHA) imbalance has been associated with chronic lung disease in CF, COPD, and asthma (Kanagaratham et al., 2014; Morin et al., 2018; Fussbroich et al., 2019; Teopompi et al., 2019).

However, the molecular mechanisms by which CFTR deficiency leads to oxidative stress, skewed bioactive lipid metabolism, and pro-inflammatory signaling have not been resolved. Moreover, the effects of available and recently approved drugs that correct the trafficking of variant CFTR channels, including lumacaftor (VX-809), tezacaftor (VX-661), and elexacaftor (VX-445), or potentiate their channel gating activity (ivacaftor, VX-770), on bioactive lipid metabolism and downstream inflammatory signaling of CF airway epithelia have not been fully investigated. Recent studies show that the reduced pro-inflammatory signaling in isolated monocytes from patients with the channel gating variant R117H CFTR is enhanced after treatment with the potentiator Ivacaftor (VX-770; Hisert et al., 2020). In contrast, inflammatory signaling was reduced in monocytes and plasma from patients homozygous for the F508del CFTR trafficking deficient variant, present in 90% of CF patients, treated with ivacaftor/lumacaftor or ivacaftor/tezacaftor (Jarosz-Griffiths et al., 2020). A general assumption is that restoring CFTR channel activity will result in the remission of all downstream defects in pro-inflammatory and tissue remodeling pathways, however, there is as yet little information on the long-term impact of CFTR modulators on abnormalities other than transepithelial Cl^−^ secretion (Rubin et al., 2019). Ruffin et al. (2018) showed that the inflammatory response of primary differentiated CF airway cells to bacterial toxins is reduced by ivacaftor/lumacaftor, dependent on the rescue of CFTR activity. However, this does not explain oxidative stress and enhanced pro-inflammatory signaling in CF epithelial cells under basal conditions reported by us previously (Stolarczyk and Scholte, 2018; Stolarczyk et al., 2018), which may contribute to the development of early CF lung disease.

Studies in CF patients show a large variation in lung pathology and inflammation parameters, may be confounded by environmental factors, and do not provide detailed information about molecular mechanisms or the contributions of different cell types involved in CF pathology. Model studies using immortalized epithelial cell lines are limited by the poor differentiation and genetic instability of cell lines, compared to human organs *in situ* and by their lack of individual variation.

We have performed mass spectrometric lipid analysis and protein arrays to study lipidomics and inflammatory markers in well-differentiated primary airway cells in air-liquid interface (ALI) from human patients carrying different CFTR variants (CF HBEC-ALI) and newborn CFTR KO pigs (Klymiuk et al., 2012; PigBEC-ALI), compared to control. This has enabled us to determine lipid and cytokine alterations in CF airway epithelial cells and responses to pharmaceutical interventions under well-controlled conditions in three independent models of CF and examine the variation between individual responses *in vitro*.

## Materials and Methods

### CF Mice and CF Pigs

Breeding of adult (80–115 days) Cftr^tm1eur^ F508del CFTR homozygous mice and sex- and age-matched normal littermates (F12 C57BL/6 backcross), isolation of lung tissue and lipid extraction for mass spec analysis was as previously described (Veltman et al., 2016). All experiments were performed with approval (DEC 138-11-09) of the Independent Committee on Ethical Use of Experimental Animals, Rotterdam, according to national and European Union guidelines. Freshly excised lungs from newborn CFTRKO Piglets and non-CF control littermates (Klymiuk et al., 2012) were obtained from the Department of Veterinary Medicine, Ludwig Maximilian University, Munich, Germany (Klymiuk et al., 2012) shipped on ice, and processed within 24 h, according to local and EU ethical guidelines.

### Human Material

*Non-CF* Human airway epithelial cells were obtained from macroscopically normal, tumor-free, anonymous bronchial tissue obtained from lung cancer patients undergoing resection surgery for lung cancer at Erasmus MC Rotterdam, approved by the Medical ethical committee of the Erasmus MC (METC 2012-512; P5, P10, P11, P24, all-male, ex-smokers 58–76 years old). All CF primary epithelial cells, five donors aged 12–32 years no history of smoking (Table 1) and one non-CF sample or (BP954) were obtained from the CF Translational Center, McGill University, Montreal, Canada according to a protocol approved by the Institutional Ethics Protocol Review Board of McGill University (#A08-M70-14B).

**Table 1:** Short circuit current responses to forskolin (Δ*I*_sc_Forsk) for well-differentiated primary bronchial epithelial cells used in this study, after treatment with correctors.

			Δ*I*_sc_Forsk (%WT)	
Donor	Gender (age)		VX-809	VX-445 + VX-661
BCF000174 (F508del/F508del)	F	(26)	16.2	n/a
BCF000554 (F508del/711+1G-T)	M	(30)	2.2	21.5
BCF000899 (F508del/I507del)	M	(32)	7.6	n/a
BCF000191 (F508del/F508del)	M	(21)	6.1	56.1
BCF000584 (F508del/F508del)	F	(12)	10.2	65.4

Non-CF data were collected from 11 donors HBEC-ALI (P1), and their mean short circuit current response to the cAMP agonist forskolin (Δ*I*_sc_Forsk, 48.4 ± 4.5 μA/cm^2^, Mean ± S.E.M) was used to calculate the average CFTR function after the rescue of CF cells expressed as % of non-CF (WT). CF HBEC ALI cells (P1, *N* = 3) were treated with lumacaftor (VX-809), 1 μM for 24 h, or elexacaftor (VX-445) plus tezacaftor (VX-661), 3 μM each for 24 h, followed by treatment with forskolin and ivacaftor (VX770) 300 nM. SEM is 10% or less of the average shown in all experiments. n/a: assay not performed for this donor.

### Cell Culture

All cell culture experiments were performed at the Erasmus MC Cell biology facility. Primary airway cells from newborn CFTR KO and control piglets and human lungs were isolated and cultured in parallel at ALI on permeable membranes to allow differentiation to airway epithelial cells (Methods BEGM) as described in Stolarczyk et al. (2016, 2018). Undifferentiated CF and non-CF primary epithelial cells frozen in liquid nitrogen were obtained from the CF Translational Center, McGill University, Montreal, Canada where they were prepared according to a protocol previously described in detail (Matthes et al., 2018). Non-CF undifferentiated primary epithelial cells from Erasmus MC were obtained stored in liquid nitrogen and cultured using essentially the same method (Amatngalim et al., 2018). In all experiments reported here CF and non-CF cells were obtained as undifferentiated primary bronchial epithelial cells, expanded submerged on collagen-fibronectin coated flasks in KSFM + ROCKinhibitor up to passage 3, and subsequently differentiated in parallel on 12 mm transwell inserts with 0.4 μl pore polyester membrane (Costar) in ALI culture (BEGM, LONZA), as described previously (Stolarczyk et al., 2016, 2018). Human and neonatal CFTR KO and WT Pig BEC-ALI cultured for 21 days, used in all experiments, showed comparable trans-epithelial resistance (range 400–800 Ω•cm^2^, *N* = 65, average 550 ± 150 SD Ω•cm^2^). Differentiated epithelial morphology as shown by lateral cadherin (ECAD) and tight junction (ZO-1) staining, and 20–30% ciliated cells (Tubulin IV) was verified in all experimental groups. Markers of secretory cells (MUC5B, MUC5AC, and SCGB3A1) were also tested under these conditions on randomly selected samples. No obvious differences between CF and non-CF were observed by visual inspection (data not shown), representative images of non-CF patients, three donors, are shown in the Supplement (Supplementary Figure S1).

### Treatment of Differentiated Cells

In all experiments reported, the differentiated cells in ALI culture were cultured on BEGM without the addition of EGF (BEGM minus EGF) for 24 h to achieve a basal state of EGFR signaling, previous to intervention or control in BEGM minus EGF (Stolarczyk et al., 2016, 2018). After a further 24 h, media were collected, an apical wash was performed, and cells were harvested for RNA isolation or lipid analysis. Fenretinide dissolved in 95% Ethanol was added to the cultures at 1,000 × stock solution to a final concentration of 1.25 μM. Reduced Glutathione (GSH, Sigma) was added as a fresh 10 mm solution in BEGM-EGF to the apical and basolateral side, the apical medium was removed after 20 min. Deferoxamine (DFO) solubilized in PBS was added to 100 μM (HBEC-ALI) or 50 μM (Pig BEC-ALI). Lumacaftor/ivacaftor (a.k.a Orkambi™) treatment was simulated by adding 2 μM VX-809 and 0.01 μM VX-770 (in 0.03% DMSO final concentration) to the cultures for 24 h. The triple compound therapy elexacaftor/tezacaftor/ivacaftor (a.k.a Trikafta™) was simulated by adding 1 μM VX-445-1 (MedChemExpress, Monmouth Junction, NJ), 3 μM VX-661, and 0.01 μM VX-770 (SelleckChem, Houston, TX; in 0.1% DMSO final concentration) for 24 or 72 h in BEGM minus EGF. Media were collected at 24, 48, and 72 h, cells were collected at 24 and 72 h. Results were compared to vehicle control experiments performed on the same day with cells from the same plating.

### Electrophysiology

Patients vary in their responses to CFTR modulators *in vitro* HBE cells (Matthes et al., 2018). To examine the impact of CFTR deficiency on lipid profiles and cytokines in cells with strong and weak drug responses, cells were studied from the CF patients used in lipid analysis, three of which are homozygous and two heterozygous for F508del-CFTR, after selection based on CFTR dependent responses in Ussing chamber experiments (Table 1). Well-differentiated monolayers were pretreated for 24 h at 37°C with vehicle (0.1% DMSO) or under conditions designed to mimic the clinically-prescribed combination drugs lumacaftor/ivacaftor (1 μM VX809 for 24 h, followed by acute treatment with 300 nM VX770 or 50 μM genistein) or ivacaftor/tezacaftor/elexacaftor (3 μM VX445-1 plus 3 μM VX661 with or without 10 nM VX770 for 24 h). Monolayers were mounted in modified Ussing chambers and short-circuit current (*I*_sc_ in μA·cm^−2^) was measured to assay of CFTR function as described previously (Matthes et al., 2018). Transepithelial voltage was clamped at 0 mV except for 2 s bipolar pulses to ±1 mV at 100 s intervals to monitor transepithelial resistance. A basolateral-to-apical NaCl chloride gradient was imposed to ensure a driving force for Cl^−^ secretion: Apical (in mM): 1.2 NaCl, 115 Na-gluconate, 25 NaHCO_3_, 1.2 MgCl_2_, 4 CaCl_2_, 2.4 KH_2_PO_4_, 1.24 K_2_HPO_4_, 10 Glucose; basolateral (in mM): 115 NaCl, 25 NaHCO_3_, 1.2 MgCl, 1.2 CaCl_2_, 2.4 KH_2_PO_4_, 1.24 K_2_HPO_4_, 10 Glucose. After the short-circuit current had stabilized (typically 2–3 min), Fsk (10 μM) was added to both sides to raise intracellular cAMP, and this was followed by sequential addition of a potentiator (50 μM genistein or 300 nM VX-770), the CFTR inhibitor CFTR_inh_-172 (10 μM) and the purinergic agonist ATP (100 μM, apical) to stimulate Ca^2+^-activated Cl^−^ channels as a positive control and to confirm cell viability (Matthes et al., 2016).

### Lipid Analysis

Cells were harvested by scraping from the insert membrane in 100 μl PBS + cOmplete Mini protease inhibitor and PhosSTOP phosphatase inhibitor (Sigma, at the concentrations indicated by the manufacturer), and the lipid fraction was extracted using 2 vol (chloroform): 1 vol (methanol): 1 mM Butylhydroxyanisol (BHA). Tubes were mixed vigorously and centrifuged at 4°C for 5 min at 3,000 rpm. The organic phase was recovered and evaporated using a Speedvac and stored in liquid nitrogen. The extracted lipids were separated as previously described (Guilbault et al., 2008, 2009; Garic et al., 2017, 2020). Malondialdehyde measurement (MDA), was performed by the TBARS assay as described previously (Guilbault et al., 2009; Garic et al., 2017, 2020). Nitrotyrosine and total ceramides were quantified as previously described in detail (Guilbault et al., 2008, 2009; Garic et al., 2017, 2020). Total ceramides were measured by ELISA after TLC purification, whereas quantification of the specific ceramides species were done using mass spectroscopy using the total ceramide pool purified on TLC. Mouse lung lipids was collected and analyzed by mass spectrometry after extraction as described in (Veltman et al., 2016).

LC-MS/MS was carried out using a QTrap 5500 mass spectrometer (AB sciex) coupled to a Dionex UltiMate 3000 LC-system. The separation column was Kinetex 2.1 × 50 mm C18, guarded with a SecurityGuard 4 × 2.0 mm C18 guard pre-column (Phenomenex). The mobile phases were MilliQ water (A) and methanol (B), both with 0.01% acetic acid, with the following gradient: first minute 20% B, increase to 35% B in 3 min and further increase to 99% B in 15 min and 100% B in 17.1. The 100% B was held for 0.9 min, then the gradient was decreased back to 20% B in 1 min and column was equilibrated at 20% B for another minute. The total run time was 20 min. MS was operated under the following conditions: the collision gas flow was set to medium, the drying temperature was 400°C, the needle voltage was −4,500 V, the curtain gas was 30 psi, ion source gas 1 was 40 psi, and the ion source gas 2 was 30 psi. Lipid standards were purchased from Sigma Aldrich and Avanti Polar Lipids.

### Protein Array Analysis

Basal media were collected 24 h after medium change (BEGM-EGF), as described and 10 analyzed on a 96×96 Fluidigm-based protein array (Olink Proteomics, Uppsala, Sweden, Array: IMMUNO/ONCOLOGY) which monitors 92 known markers of inflammation and tissue remodeling (Supplementary Table S1). Data are presented as NPX, a normalized protein expression value, which is a unit on a log 2 scale, allowing quantitative comparison of every marker in different samples, i.e., an NPX difference of +1 represents a two-fold difference in marker concentration. Note that this does not allow a direct comparison of antigen concentrations of different markers (Olink data analysis).

*Statistical analysis and graph preparation* were performed with PRISM8 software in compliance with the PRISM8 statistics guide, and with standard procedures set by Erasmus MC biostatisticians, tests used are specified in the legends.

## Results

### Oxidative Stress and Abnormal Lipid Profile in Human and Pig CF Primary Bronchial Cells

**Figure 1:**
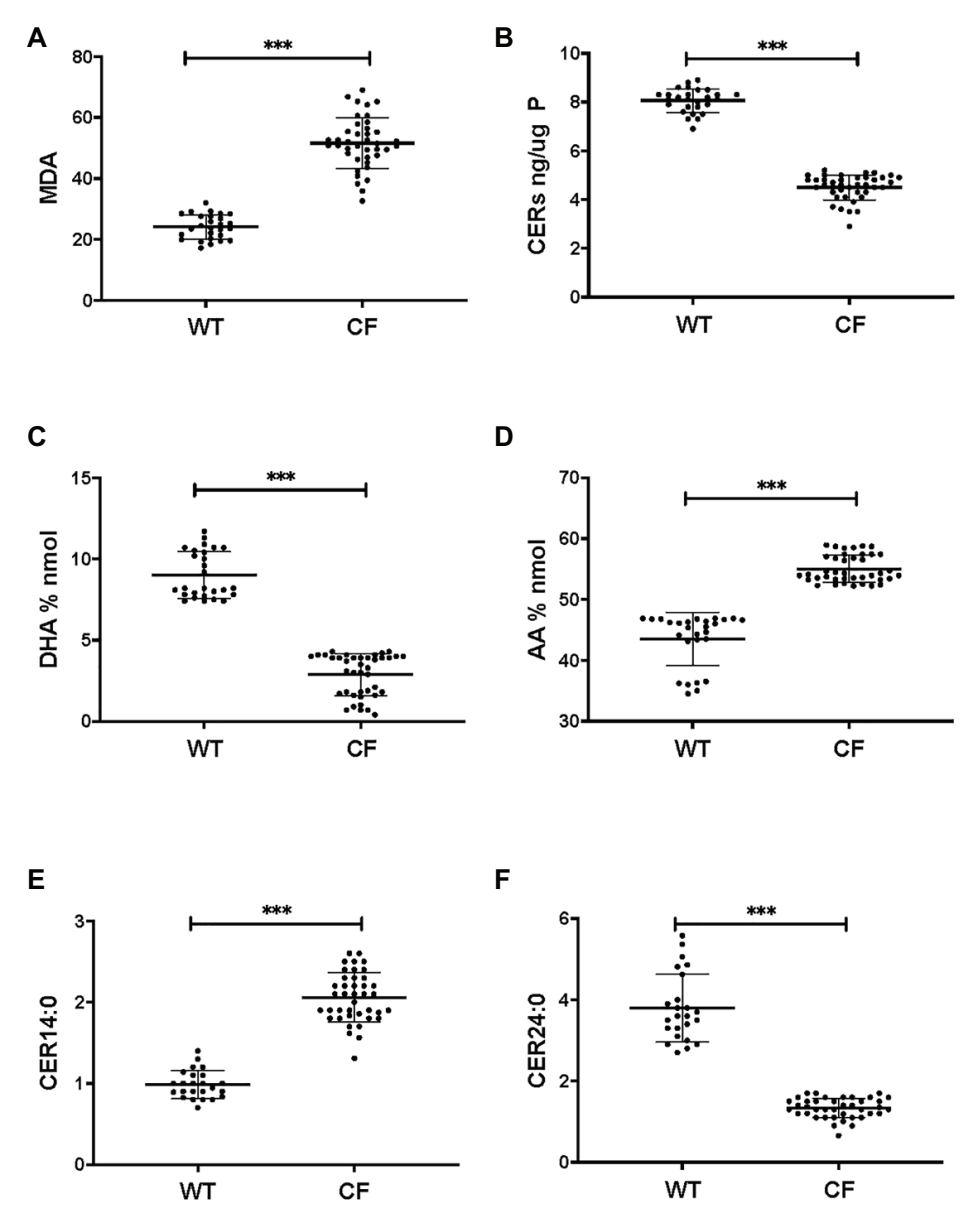
Oxidative stress, ceramides, and abnormal lipid metabolism in CF human bronchial epithelial cells in ALI culture. CF HBEC-ALI from five different donors (Table 1) compared to non-CF (WT, five different donors, see methods), were analyzed as described in methods. Data were merged from three separate experiments, comparing each donor in triplicate or quadruplicate in every experiment, data points are shown representing a single membrane (CF *N* = 40 filters, non-CF *N* = 25 filters). Horizontal bars represent average, error bars ± SD. **(A)** malondialdehyde (MDA) pmol/nmol fatty acid. **(B)** Total ceramides in CF HBEC-ALI. **(C)** Docosohexaenoic acid (DHA) levels (% nmol total fatty acids) are strongly reduced in CF HBEC-ALI compared to WT. **(D)** arachidonic acid (AA) levels (% nmol total fatty acids). **(E)** Long-chain ceramide (CER14:0). **(F)** Very long-chain ceramide (CER24:0) expressed as pmol/nmol phosphate. Statistical analysis (unpaired *T*-test) was performed with grouped averages (CF vs. Non-CF) for each different donor.

**Figure 2:**
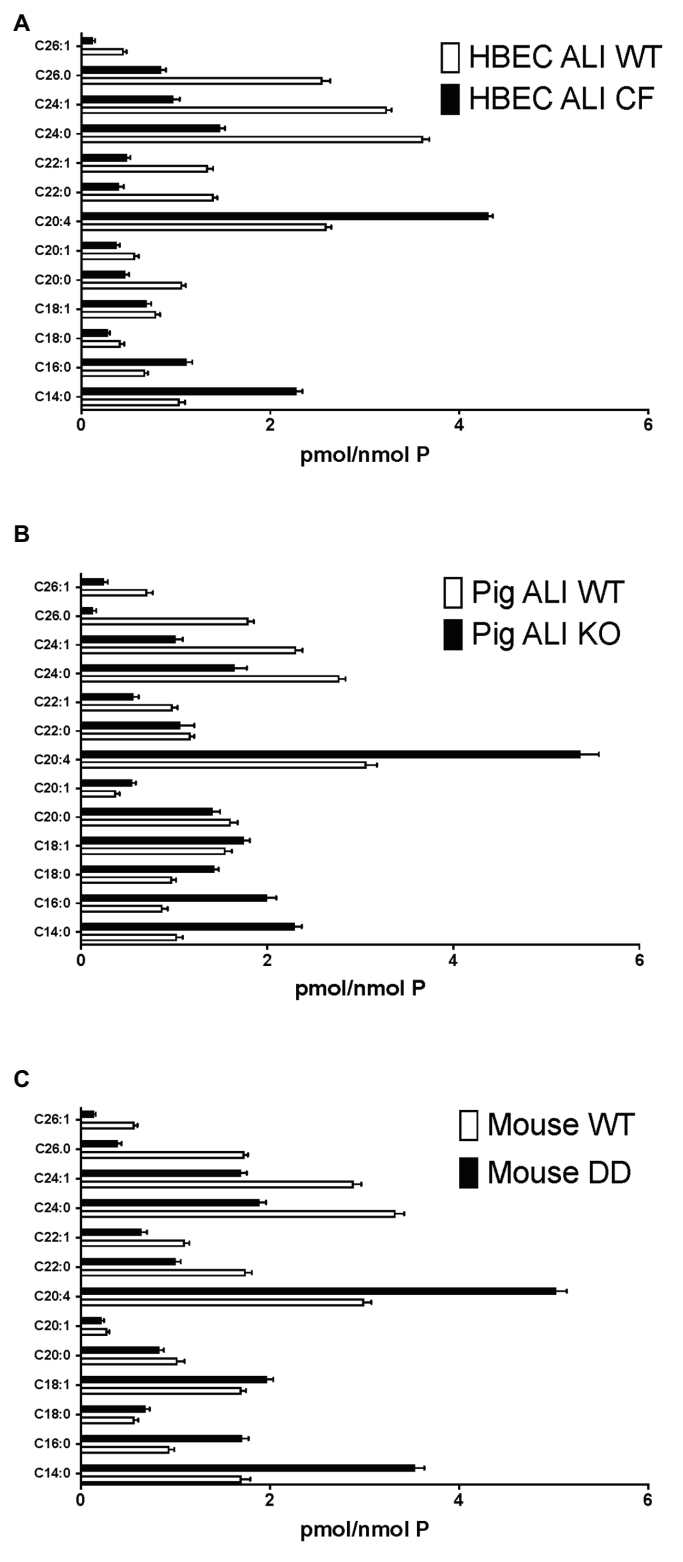
Enhanced long-chain to very long-chain ceramide species ratio in CF airway cells and lung tissue. Lipids were extracted and analyzed by mass spectrometry, as described in methods merged and averaged data. The bars represent the average concentration of individual ceramide species expressed as pmol/nmol total lipid phosphate (pmol/nmol *P*, error bars: SEM). **(A)** Human bronchial epithelial cells in ALI culture (HBEC-ALI) CF (BCF000174; *N* = 12 inserts) compared to WT (*N* = 12, three donors, four filters each), data merged from three independent experiments. Four other CF donors (Table 1) give similar results to BCF000174 as shown in (Figures 1E,F). **(B)** Pig bronchial epithelial cells in ALI culture (PIG ALI) on membrane inserts cystic fibrosis transmembrane conductance regulator (CFTR) KO (*N* = 6) and WT littermate (*N* = 9). **(C)** Total lung of age- and sex-matched adult homozygous mutant F508del CFTR (DD; *N* = 19) or control (WT) littermate mouse (*N* = 15), as described in methods.

Firstly, we applied lipid analyses to well-differentiated primary bronchial epithelial cells from five CF patients having variable corrector responses (Table 1), and five non-CF donors (methods) cultured in parallel under identical conditions. The oxidative stress and lipid peroxidation marker MDA was elevated in all CF patients compared to non-CF controls (Figure 1A). Total TLC purified ceramide levels were reduced in CF HBEC-ALI (Figure 1B). The ω-3-PUFAs DHA (Figure 1C) and related EPA (not shown) were strongly reduced whereas the most prevalent ω-6 PUFA AA was increased (Figure 1D). LCC species CER14:0 was enhanced (Figure 1E), whereas the VLCC species CER24:0 was reduced relative to control (Figure 1F). This illustrates an overall increase of LCC over VLCC levels, expressed as pmol/nmol lipid phosphate, in CF HBEC-ALI across all LCC (CER14:0, CER16:0) and VLCC (CER22:0, CER24:0, CER26:0) ceramide species (Figure 2A). The apparent exception of the CER20:4 ceramide species which carries the AA chain (20:4), may be related to the enhanced levels of this PUFA in CF cells (Figure 1D). The ceramide precursors sphingosine and dihydroceramide (DH) were also reduced in CF HBEC-ALI under all conditions examined (Supplementary Figures S2A,B).

**Figure 3:**
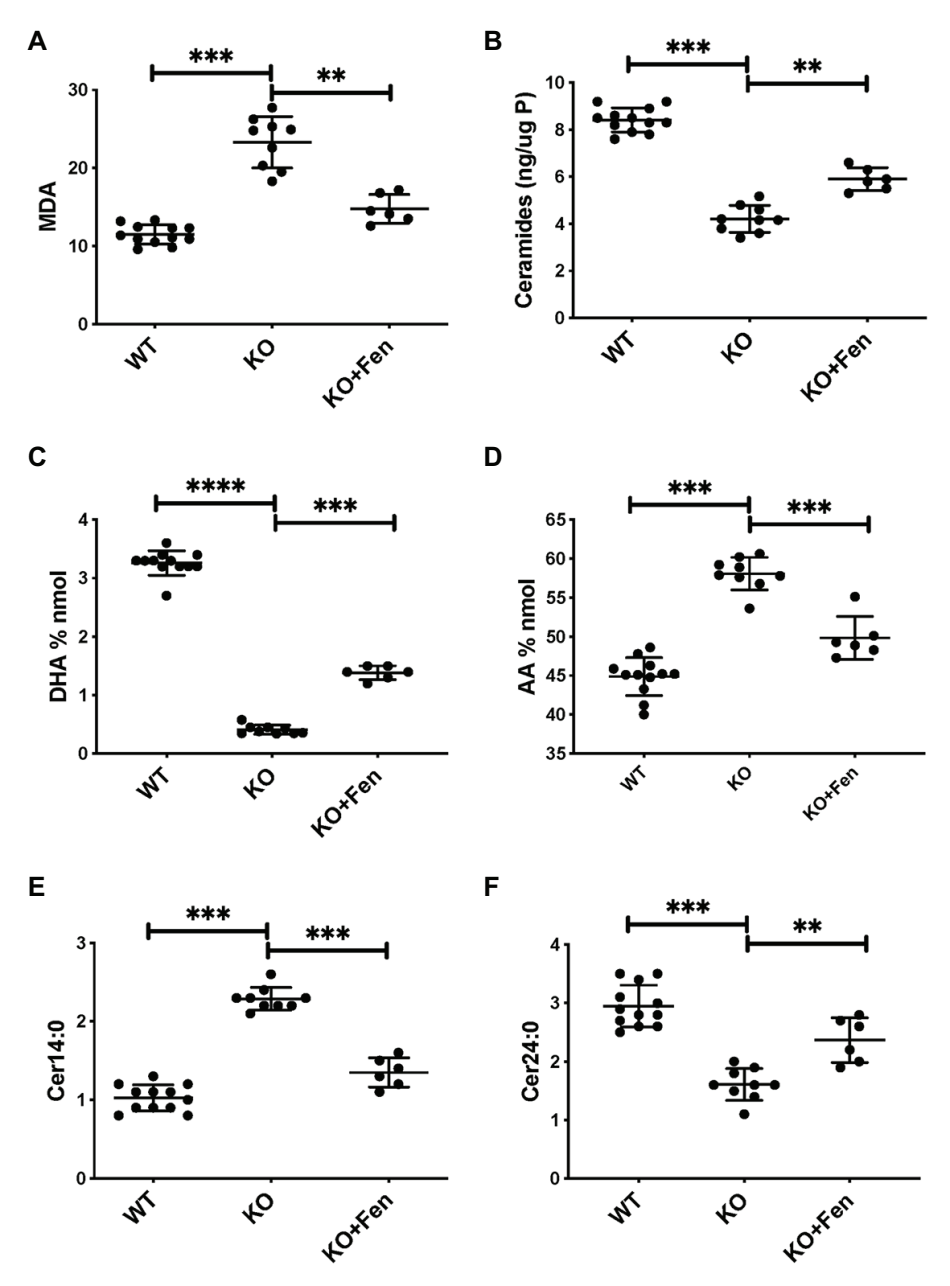
Oxidative stress and abnormal lipid balance in CFTR KO PIG bronchial cells in air-liquid interface (ALI) culture is partially corrected by Fenretinide. **(A)** malondialdehyde (MDA) pmol/nmol fatty acid. **(B)** Total ceramides in CF HBEC-ALI. **(C)** DHA levels and **(D)** AA levels (% nmol total fatty acids). **(E)** Long-chain ceramide (CER14:0) and **(F)** Very long-chain ceramide (CER24:0) expressed as pmol/nmol phosphate. All parameters show significant differences when comparing CFTR KO (*N* = 9) to WT (*N* = 12), and CFTR KO filters treated with fenretinide (*N* = 6) in parallel experiments (****Padj < 0.001, unpaired ANOVA, Dunnet, using KO as reference control). Results are from two CFTR KO and two wildtype newborn pigs cultured in parallel in two separate experiments. Symbols in the figures represent data from single filters, average ± SD.

To further establish whether lipid abnormalities are an inherent property of CF bronchial epithelia independent of their origin or culture conditions and species we analyzed the lipid profiles of well-differentiated primary bronchial epithelial cells from neonatal CFTR KO pig bronchial cells and littermate control (WT). In CF pig BEC-ALI, very similar to human CF HBEC-ALI, we observed elevated levels of MDA (Figure 3A), reduced total ceramide levels (Figure 3B). LCC species (Figure 3D) were increased whereas VLCC species were decreased (Figure 3E) in CFTR KO pig as in CF mouse lung tissue, resulting in a four-fold increase in the ratio of LCC to VLCC (Figure 2B). The ceramide precursors sphingosine and DH were reduced in CF CFTR KO pig airway cells (Supplementary Figures S2C,D) similar to CF HBEC-ALI cells mentioned above (Supplementary Figures S2A,B). Finally, the vitamin A analog fenretinide, which has previously been shown to correct the LCC/VLCC ratio in immortalized CF cell models (Garic et al., 2017) and CFTR KO mice (Youssef et al., 2020c), partially normalized all lipid levels in KO pigs towards those observed in WT pigs. The oxidative stress marker MDA (Figure 3A) and total ceramides (Figure 3B) in airway cells from CFTR KO pig were reduced and increased, respectively, DHA and AA levels were both corrected significantly (Figures 3C,D), and abundance of the LCC Cer14:0 and VLCC Cer24:0 were largely normalized (Figures 3E,F), as were the levels of ceramide precursors (Supplementary Figures S2C,D).

### Oxidative Stress Markers and Abnormal Lipid Profile in CF Mouse Lung

**Figure 4:**
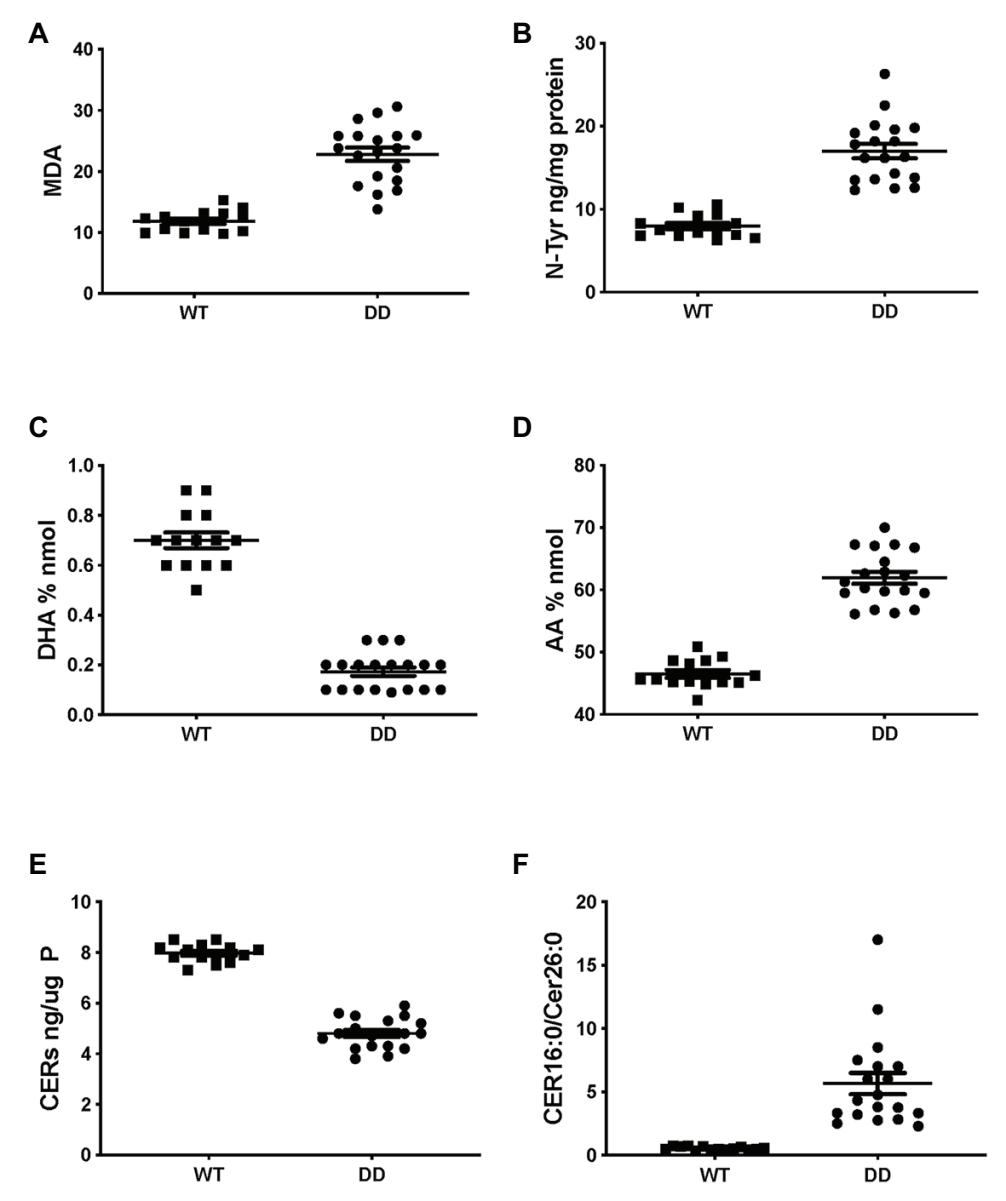
Oxidative stress and abnormal lipid levels in F508del CFTR variant mouse lung. Total lungs from adult male and female mice, homozygous for the F508del CFTR allele (DD, *N* = 19) or age- and sex-matched normal littermates (WT, *N* = 14), were extracted and lipids were analyzed as described, each data point represents a single individual. **(A)** malondialdehyde (pmol/nmol fatty acid). **(B)** Nitro-tyrosine. **(C)** DHA. **(D)** AA. **(E)** Total ceramide species after TLC purification. **(F)** LCC(Cer16:0)/VLCC(Cer26:0) ratio (compare Figure 2C). CF and WT lungs differed significantly in all parameters (unpaired *T*-test *p* < 0.001).

Very similar abnormalities in lipid levels were observed in CF mouse lung as in CFTR KO pig and human CF HBEC-ALI. Two markers of oxidative stress, MDA and nitrosylated tyrosine (N-Tyr) were elevated in adult F508del CFTR mouse lung (Figures 4A,B). There was also a marked reduction in the major ω-3 PUFA DHA 22:6 and increased levels of the ω-6 PUFA AA (Figures 4C,D), resulting in a more than 10-fold decrease in the DHA/AA ratio. These observations are consistent with our previous findings of reduced sphingosine-1-phosphate levels, sterile lung inflammation in these homozygous F508del CFTR mice (Cftr^tm1EUR^ BL/6; Veltman et al., 2016). CF mice of this strain showed mild intestinal disease and were fed normal chow like their normal (WT) littermate controls, therefore abnormal lung lipid ratios are unlikely to reflect a diet-related bias. The total amount of ceramide species quantified by ELISA after TLC purification was reduced in CF mouse lung (Figure 4E), and there was a marked increase in the ratio of LCC (Cer16:0) to VLCC (Cer26:0; Figure 4F). The complete averaged profile of ceramide species confirmed this trend towards very long acyl chains in homozygous F508del CFTR mice relative to wild type (compare with Figure 2C).

### Partial Correction of Lipid Metabolism by CFTR Targeted Therapeutics

**Figure 5:**
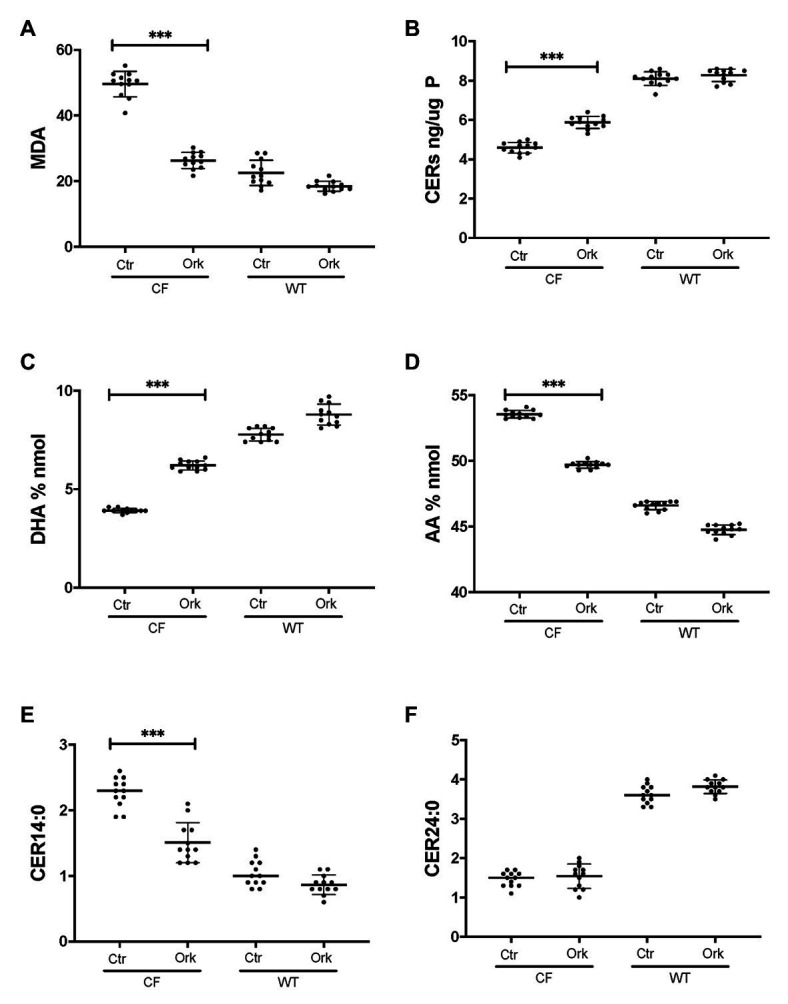
Partial correction of oxidative stress and lipid imbalance in CF HBEC-ALI by ivacaftor/lumacaftor. Treatment of the high responder (Table 1) homozygous F508del CFTR BCF174 HBEC ALI with VX-809 (2 μM) and VX-770 10 nM for 24 h (Ork) causes partial correction of lipid imbalance, compared to carrier control (Ctr; *N* = 12). Non-CF (WT) cells are represented by three donors in quadruplicate each (*N* = 12). **(A)** malondialdehyde (MDA) pmol/nmol fatty acid. **(B)** Total ceramides in CF HBEC-ALI. **(C)** DHA levels (% nmol total fatty acids). **(D)** AA levels (% nmol total fatty acids). **(E)** Long-chain ceramide (CER14:0). **(F)** Very long-chain ceramide (CER24:0) both expressed as pmol/nmol phosphate. Most relevant comparisons are indicated (unpaired ANOVA, Dunnet, *** *p* < 0.001, untreated CF as control).

To examine if CFTR functional correction can normalize lipid metabolism we treated HBEC-ALI cells from an F508del homozygous patient (BCF000174) with VX-809 and VX-770 to mimic the clinically approved combination drug ivacaftor/lumacaftor (a.k.a Orkambi). These CFTR modulators enhanced CFTR activation by forskolin in Ussing chamber and increased apical fluid height up to 25% of WT levels, which is considered a robust response compared to most other CF populations tested and expected to provide significant clinical benefit to patients (Table 1). The low response of BCF000554 may reflect a gene dose effect since that patient had a splice site mutation at one allele (711 +1G-T) and hence would have only about half the number of CFTR transcripts compared to F508del homozygous cells. I507del causes a folding and trafficking defect very similar to F508del, which is consistent with the level of correction observed. While useful in acute stimulation, chronic exposure to high concentrations of ivacaftor (VX-770). In cell culture models, reduces correction of F508del-CFTR by lumacaftor (VX-809; Cholon et al., 2014; Veit et al., 2014; Matthes et al., 2016). However, inhibition of functional rescue by ivacaftor is negligible at clinically-relevant concentrations at 1–10 nM (Matthes et al., 2016; Csanady and Torocsik, 2019). Treatment with 10 nM ivacaftor and lumacaftor (2 μM) for 24 h, reduced levels of the oxidative stress marker MDA towards normal values in CF HBEC-ALI (Figure 5A). However, total ceramide (Figure 5B), DHA and AA levels (Figures 5C,D), and LCC ceramide (CER14:0, Figure 5E) were only partially corrected. VLCC (CER24:0) was not significantly affected (Figure 5F). Non-CF (WT) cell populations (compare Figure 1) served as parallel controls; the data range (average ± SEM) of the parallel controls is indicated by a hatched bar, for comparison.

**Figure 6:**
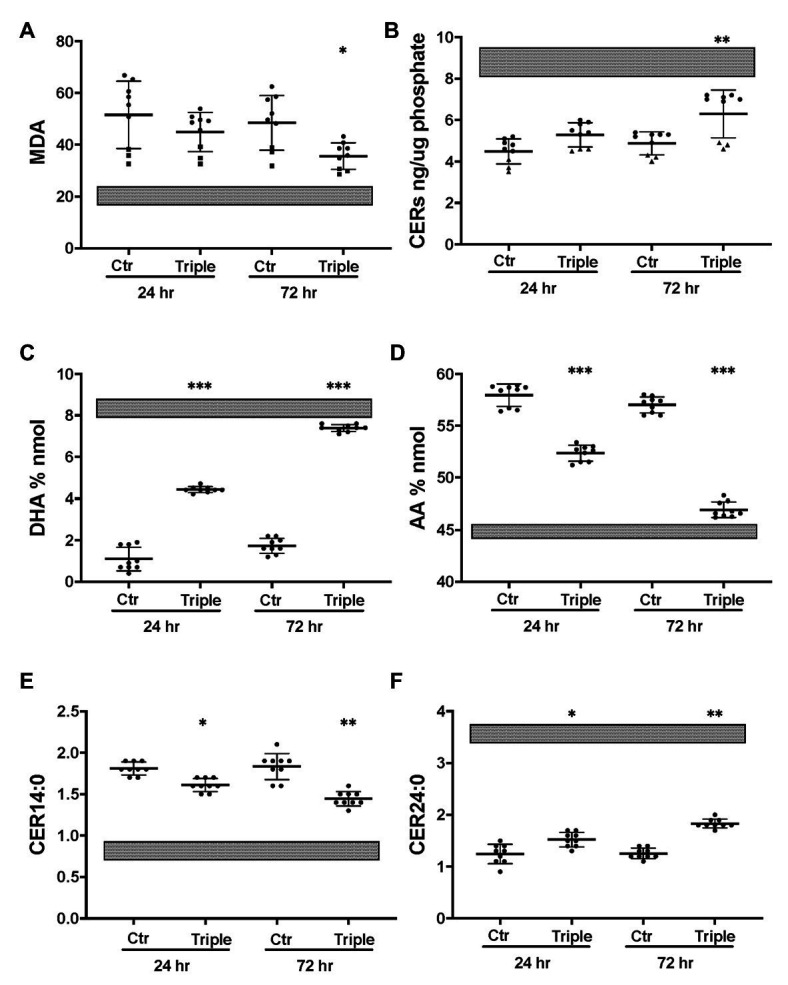
Partial correction of oxidative stress and lipid imbalance in CF HBEC-ALI by elexacaftor/tezacaftor/ivacaftor triple therapy. CF HBEC ALI were treated for 24 or 72 h with the triple combination elexacaftor/tezacaftor/ivacaftor (VX-445 + VX-661 + VX-770; Triple) or vehicle alone (Ctr). CF HBEC ALI inserts of three donors, defined by their rescue by ivacaftor/lumacaftor and in Ussing chamber experiments Medium responder (BCF000191) low responder (BCF000554, squares) and high responder (BCF000584, triangles; Supplementary Table S1). Filters were analyzed in triplicate at every time point, data points (*N* = 9) represent single inserts. Avarage Levels ± SEM are shown after treatment with elexacaftor/tezacaftor/ivacaftor or control vehicle (Ctr) for 24 or 72 h. Treatment of Non-CF HBEC in parallel with elexacaftor/tezacaftor/ivacaftor had no detectable effect at either timepoint (WP945, *N* = 6, not shown). For comparison, gray bars represent average ± SEM of parallel Non-CF data obtained in parallel (three donors, *N* = 12). **(A)** malondialdehyde (MDA) pmol/nmol fatty acid. **(B)** Total ceramides. **(C)** DHA levels (% nmol total fatty acids). **(D)** AA levels (% nmol total fatty acids). **(E)** Long chain ceramide (CER14:0). **(F)** Very long chain ceramide (CER24:0) both expressed as pmol/nmol phosphate. Most relevant comparisons are indicated (unpaired ANOVA, Dunnet, ^*^Padj < 0.05, **p < 0.01, ***p < 0.001, using parallel carrier treated control at 24 and 72 h, respectively, as control).

The triple combination treatment ivacaftor/tezacaftor/elexacaftor which consists of the correctors elexacaftor (VX-445) and tezacaftor (VX-661) plus the CFTR activator ivacaftor VX-770, is more efficacious than ivacaftor/lumacaftor in improving clinical symptoms, and is also effective in heterozygotic patients (Keating et al., 2018; Bear, 2020). These clinical findings were confirmed *in vitro* under our Ussing chamber conditions (Table 1). We next examined the effects of ivacaftor/tezacaftor/elexacaftor on the lipid profile and increased the treatment period from 24 to 72 h in case the process of adaptation and correction of lipid levels is slow compared to the rescue of F508del CFTR variant protein. We compared cells from three different patients that had been categorized as high, medium, or low responders based on their responses to ivacaftor/lumacaftor in Ussing chambers (Table 1). Ivacaftor/tezacaftor/elexacaftor reduced MDA levels and increased total ceramide abundance in CF HBEC-ALI in a donor- and time-dependent manner (Figures 6A,B). There was a gradual correction of DHA and AA to near-normal levels in all donors (Figures 6C,D). By contrast, CER14:0 and CER24:0 (Figures 6E,F), and by inference, the LCC/VLCC ratio (not shown) were only partially normalized. These results demonstrate that oxidative stress and anomalous lipid levels are CFTR dependent and can be at least partially corrected by currently available CF therapeutics. We did not observe a clear correlation between individual variation in CFTR functional correction in Ussing chambers (Table 1) and correction of the lipid imbalance in this experimental paradigm.

### Anti-oxidant Treatment Corrects Oxidative Stress and Lipid Metabolism

**Figure 7:**
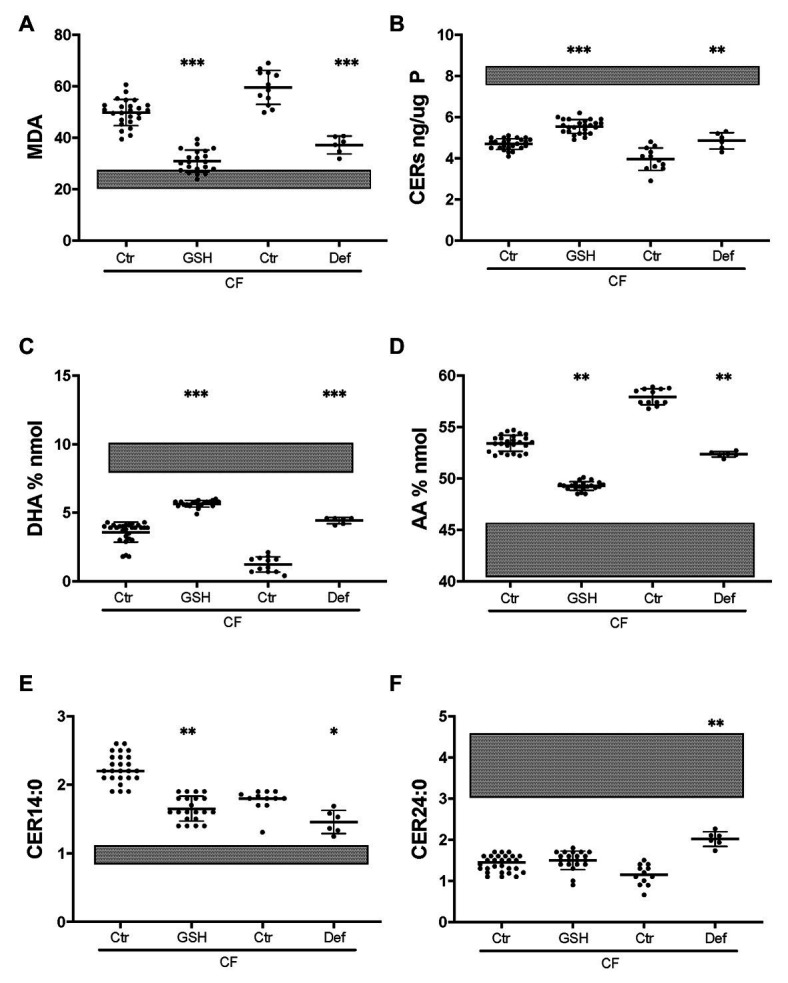
Partial correction of oxidative stress and lipid imbalance by anti-oxidants in CF HBEC-ALI. Treatment of CF HBEC ALI with extracellular glutathione (10 mM GSH, three different donors (BCF000174, *N* = 15; BCF000554, *N* = 3; BCF000889. *N* = 3; *N* = 21), or deferoxamine (Def, BCF000191 *N* = 3, BCF000584 *N* = 3) for 24 h, compared to carrier treated parallel controls of the same donors (Ctr), causes partial correction towards non-CF untreated values indicated as shaded bars representing untreated non-CF average ± SD (five donors analyzed in parallel, Figure 1). **(A)** malondialdehyde (MDA) pmol/nmol fatty acid. **(B)** Total ceramides. **(C)** DHA levels (% nmol total fatty acids). **(D)** AA levels (% nmol total fatty acids). **(E)** Long-chain ceramide (CER14:0). **(F)** Very long-chain ceramide (CER24:0) both expressed as pmol/nmol phosphate. Most relevant comparisons are indicated (unpaired one-way ANOVA, Dunnet, *p < 0.05, **p < 0.01, ***p < 0.001).

To establish whether CF-related oxidative stress contributes to abnormal lipid metabolism, the basal medium was supplemented with reduced GSH as described previously (Stolarczyk et al., 2018). Exogenous GSH caused a substantial decrease in the oxidative stress marker MDA in CF HBEC-ALI, towards normal values (Figure 7A) whereas there was no significant effect on non-CF cells (not shown). GSH supplementation partially corrected ceramide, DHA and AA levels (Figures 7B,C,D) bringing them closer to those in non-CF cells. LCC (CER14:0), was also partially corrected whereas CER24:0 was not (Figures 7E,F). The certified drug and Fe chelating antioxidant DFO had a similar effect in both CF pig (not shown) and human CF bronchial epithelial cells (Figure 7). These data suggest that lipid imbalance in primary bronchial epithelia is, at least in part, due to CFTR-related oxidative stress and can be corrected by anti-oxidant treatment.

### Oxidative Stress and Lipid Imbalance are Associated With Enhanced Pro-Inflammatory Signaling

**Figure 8:**
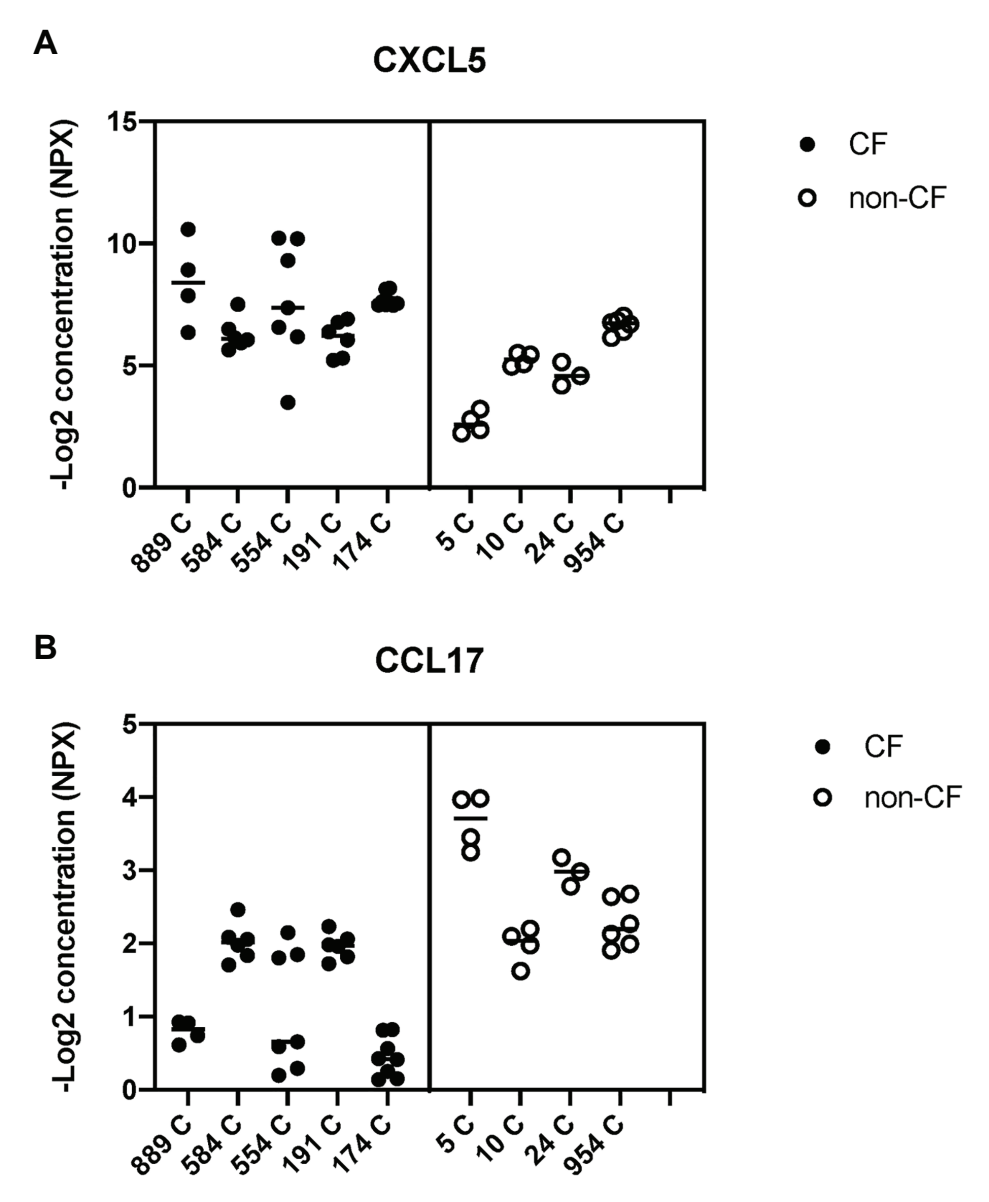
Basal shedding of cytokines and growth factors by primary CF and non-CF airway epithelial cells. Markers of inflammation and tissue remodeling were measured by a Fluidigm-based protein array (Olink 96×96 Oncology/inflammation) in basal medium (BEGM minus EGF), 24 h after the medium change. CF HBEC-ALI (five donors, Table 1, 31 samples) compared to non-CF (WT) cultured in parallel (four donors, 17 samples). Complete array data are summarized in Supplementary Table S1. Data are expressed as NPX (−Log2 expression relative to the internal control). **(A)** CXCL5 (C-X-C chemokine ligand 5) involved in neutrophil activation and angiogenesis is on average four-fold (two logs) higher in CF media compared to non-CF. **(B)** CCL17 (C-C motif chemokine ligand 17) involved in trafficking and maturation of T-cells is four-fold lower in CF HBEC [statistical analysis on total merged array data by multiple *T*-test *p* < 0.001, *q* < 0.001 (Supplementary Table S2), and nested *T*-test of donor-specific data (*p* < 0.02) shown here].

To study alterations in cytokines and growth factors in well-differentiated CF vs. non-CF bronchial epithelial cells, a 96×96 microfluidics-based protein array focused on markers of inflammation and tissue remodeling was used. Secretion of 41 cytokines and growth factors was detected by analyzing the basal medium of CF and non-CF primary epithelial cells (Supplementary Table S1). Twelve of these proteins were altered significantly in CF compared to non-CF cultures (Table 2). In particular, the neutrophil-activating chemokine CXCL5 is upregulated whereas the T-cell activator CCL17 is reduced in CF HBEC-ALI (Figure 8).

**Table 2:** Shedding of cytokines and growth factors by primary CF epithelial cells compared to non-CF.

	% WT	SEM	*P* adj	*q*
**CXCL5**	771	351	<0.000	<0.000
**PGF**	453	195	<0.000	0.001
**HO-1**	429	291	0.008	0.019
**IL18**	429	251	0.003	0.014
**Gal-1**	416	249	0.005	0.014
**ADA**	219	64	0.004	0.014
**CAIX**	215	66	0.007	0.018
**CXCL11**	214	60	0.004	0.014
**CASP-8**	213	53	0.002	0.010
**TNFRSF21**	49	9	<0.000	0.001
**CXCL1**	49	15	0.012	0.025
**CCL17**	28	6	<0.000	<0.000

Relative marker concentration in CF HBEC ALI basal medium compared to non-CF, expressed as percentage of average non-CF (%WT, ±SEM) was analyzed using Fluidigm™ based protein array analysis (Olink 96×96 Oncology/inflammation), 24 h after medium change (BEGM-EGF), merged data from a single array. CF HBEC-ALI (five donors, 22 samples) compared to non-CF (four donors, 17 samples). Statistical analysis by multiple *T*-test (discovery defined as more than two-fold change and *p* < 0.05; *q* < 0.05, was determined using the two-stage linear step-up procedure of Benjamini, Krieger, and Yekutieli, without assuming consistent SD).

To examine the role of CFTR using a pharmacological approach, well-differentiated HBEC-ALI cells were pretreated with ivacaftor/lumacaftor or DFO for 24 h. As shown above, both treatments partially corrected the oxidative stress and lipid imbalance, however, neither agent altered cytokine output significantly in CF or non-CF cells (*n* = 3 patients, *N* = 4 each, data not shown). Treatment with the more potent corrector combination drug elexacaftor/tezacaftor/ivacaftor for 24–72 h, caused modest reductions in levels of the growth factor PFG and pro-inflammatory cytokine CCL20 in all three patients but did not reduce CXCL5, or CXCL11 significantly (Supplementary Figure S3).

By contrast, GSH supplementation did reduce the shedding of pro-inflammatory and pro-fibrotic factors CCL20, CSF-1, and MCP-1, more than 10-fold in CF HBEC-ALI (three donors, 21 samples, not shown). CXCL5 and several other factors were excluded from the analysis because they could not be measured reliably in the presence of the reducing agent GSH. In summary, partial correction of the oxidative stress and abnormal lipid profile by clinically approved CF drugs had only modest effects on pro-inflammatory cytokine production.

## Discussion

The present results extend our previous studies of CF infant BALF and CF mouse tissues and clarify the relationship between CFTR deficiency, oxidative stress, and abnormal lipid levels.

In this study, we have shown that CFTR deficient mice and well-differentiated bronchial epithelial cultures from two different species display similar abnormalities and that they vary in their responses to clinically approved CF drugs. Several lipids measured in this paper are bioactive modulators of lung inflammation and tissue remodeling which interact with multiple signaling pathways, are likely to have a causal role in CF pathogenesis, and are potential therapeutic targets. The vitamin A analog fenretinide corrects the high ω-6/ω-3 (AA/DHA) fatty acid ratio (Guilbault et al., 2009), the ratios of LCC/VLCC ceramide species and mucus production in CFTR KO mouse lung, and plasma lipid imbalance in CF patients (Garic et al., 2017, 2020; Youssef et al., 2020c). Fenretinide also normalizes fatty acid ratios and reduces lung inflammation in a mouse model of allergic asthma (Kanagaratham et al., 2014; Youssef et al., 2020a,b). Amitriptyline, an inhibitor of an acid sphingomyelinase, is reported to improve lung function and body weight in CF patients (Adams et al., 2016). In the present study, we also showed enhanced lipid peroxidation, combined with enhanced ω-6 to ω-3 PUFA (AA/DHA) ratio, and ceramide species (LCC/VLCC) ratio in total lung extracts in this model. In CFTR KO mice with more severe intestinal pathology, we recently observed that the LCC/VLCC ratio and age-dependent lung pathology were both corrected by the retinoic acid analog fenretinide (Youssef et al., 2020c). Taken together, the results suggest that both CF mouse and infant CF airways lungs present with an inherently abnormal pro-inflammatory milieu due to elevated oxidative stress and abnormal lipid metabolism even before they become infected. The primary source of this skewed signaling in the CF lung remains to be elucidated.

The results indicate there is elevated oxidative stress, an abnormal lipid fingerprint, and enhanced pro-inflammatory signaling in well-differentiated bronchial epithelial cells isolated from the neonatal CFTR KO pigs and from adult CF patients undergoing lung transplantation. Overall, the oxidative stress markers lipid peroxidation and protein nitrosylation correlated with abnormal levels of essential fatty acids and sphingolipids and altered ceramide metabolism in three different models of CF; F508del CFTR mouse lung, CFTR KO Pig, and CF human epithelial cells in culture. The present data further show that these abnormalities are associated with enhanced basal cytokine and growth factor shedding and changes in gene transcription. In previous studies, we have shown that enhanced activity of the EGFR/ADAM17 axis in CF HBEC-ALI, which controls the shedding of multiple growth factors and cytokines, is tightly regulated by extracellular redox signaling (Stolarczyk and Scholte, 2018).

Despite the different origins of the human primary cell populations, the CF and non-CF lipid data show little variation within each group and no overlap between the groups. Moreover, CFTRKO PIG airway epithelial cells and CF mouse lung show a very similar signature. We conclude therefore that oxidative stress, lipid imbalance, and enhanced pro-inflammatory and tissue remodeling signaling are cell-autonomous features of well-differentiated primary CF airway epithelia.

Future quantitative proteome and transcriptome analysis in our model can establish which specific pathways and enzymes are affected by CFTR deficiency. A preliminary RNAseq analysis of BCF000174 compared to two non-CF donors (WP11, WP21) cultured and analyzed in parallel confirms the expression of all enzymes known to be involved in ceramide synthesis, including ceramide synthase isozymes, PUFA elongases (ELOVL), and fatty acid desaturases (FADS), but the data do not readily explain the observed lipid imbalance in CF HBEC-ALI (Scholte et al., 2021). Most likely, the changes in ceramide and PUFA metabolism are caused, at least in part, by translational and posttranslational events such as trafficking and enzyme compartmentalization which, could alter metabolic flux and steady-state levels of ceramides and PUFAs. Using a pulse labeling technique to quantitate sphingolipids, Loberto et al. (2020) recently reported enhanced ceramide and ganglioside levels specifically in the apical membrane of CF compared to non-CF in primary airway epithelial cells, differentiated 7 days at the ALI, associated with a change in relative localization of sphingolipid metabolizing enzymes.

### Fenretinide Corrects Oxidative Stress and Lipid Imbalance in Airway Epithelial Cells

As discussed below, several pharmaceutical interventions with different molecular targets were found to normalize CF lipids. Oxidative stress, fatty acid, and ceramide metabolism were partially corrected by fenretinide in CFTR KO pig primary airway cells, extending recent data confirming its involvement in ceramide metabolism obtained from CFTR KO mice (Youssef et al., 2020c) and CF model cell lines (Guilbault et al., 2009; Garic et al., 2017). Fenretinide has anti-inflammatory (Kanagaratham et al., 2014; Orienti et al., 2020) and pro-apoptotic activities (Anding et al., 2018). However, its molecular mechanisms are complex, involving both retinoic acid receptor (RAR)-dependent and -independent pathways. Fenretinide modulates lipid turnover, autophagy, apoptosis, and mitochondrial function (McIlroy et al., 2016), which are affected by epithelial CFTR deficiency. It is also noteworthy that fenretinide is a selective inhibitor of DEGS1, a sphingolipid desaturase involved in the production of ceramides from DH (Rahmaniyan et al., 2011). The DEGS1 and parallel DEGS2 pathways may be involved in controlling LCC/VLCC ratios and have been proposed as a sensor of metabolic oxidative stress (Devlin et al., 2011). Total DH was low and the LCC/VLCC ratio was increased in bronchial epithelial cells from CF pigs, and both were partially corrected by fenretinide consistent with an upregulation of DEGS1 activity. This would provide a molecular basis for the reduced DH levels and the enhanced LCC/VLCC ratio in CF HBEC-ALI, and the effect of fenretinide on this parameter.

### CF Related Oxidative Stress Drives Lipid Imbalance

The molecular mechanism that links CFTR deficiency with oxidative stress has not been firmly established. ER stress due to defective processing of F508del CFTR can be excluded as it is observed in cells from CFTR knockout pigs and mice that are devoid of CFTR. Several studies have invoked abnormal GSH homeostasis (Dickerhof et al., 2017; de Bari et al., 2018) or deficient membrane trafficking and autophagy (Esposito et al., 2016), and mitochondrial dysfunction (Kleme et al., 2018) as sources of cellular stress in CF. While all these mechanisms may contribute, they do not yet provide a definitive explanation for how the dysfunction of an apical chloride channel leads to the complex abnormal lipid phenotype reported here.

Deferoxamine and GSH supplementation reduced oxidative stress in CF HBEC-ALI and partially normalized PUFA, sphingosine, and ceramide metabolism, suggesting a causal link between CFTR deficiency-related oxidative stress and lipid imbalance. These maneuvers have different molecular targets. DFO is an approved drug that is used to treat Fe overload toxicity in thalassemia and sickle-cell disease. It reportedly has anti-oxidant properties and reduces lipid peroxidation and cell injury in an acute model of cigarette smoke exposure (Yoshida et al., 2019), and improves tissue regeneration (Vlahakos et al., 2012; Holden and Nair, 2019). Interestingly, Fe accumulation associated with lung pathology has been observed in CF lung (Ghio et al., 2013). GSH is a well-known antioxidant in the lung (Cantin et al., 1987) that can be transported by CFTR (Linsdell and Hanrahan, 1998; Kogan et al., 2003). Although we cannot exclude an effect of antioxidants on CFTR activity or an antioxidant effect of the CFTR modulators in human CF cells, we did not observe a significant effect of these interventions on lipid parameters in non-CF cells (data not shown). Therefore, the results suggest that epithelial oxidative stress caused by CFTR deficiency, likely involving abnormal Fe homeostasis, alters lipid metabolism which might be partially normalized by anti-oxidant treatment *in vivo* (Holden and Nair, 2019).

### CFTR Targeted Therapeutics Partially Correct Oxidative Stress and Lipid Imbalance

Treatments that mimic ivacaftor/lumacaftor and the more potent triple combination therapy elexacaftor/tezacaftor/ivacaftor significantly reduced lipid peroxidation but did not normalize it completely. Ceramide, sphingosine, DH levels, and LCC/VLCC ratio were only partially corrected to a similar extent by both treatments. DHA and AA levels were substantially corrected, 72 h after elexacaftor/tezacaftor/ivacaftor treatment. The effect of elexacaftor/tezacaftor/ivacaftor was more pronounced after 72 h compared to 24 h in all parameters, suggesting a relatively slow process of adaptation at the transcriptional and or posttranscriptional level following CFTR rescue. This is consistent with the involvement of CFTR function in regulating oxidative stress and lipid metabolism, considering the significant effect of these compounds on F508del CFTR cAMP-induced chloride transport function in Ussing chamber experiments within 24 h. Using a different approach in submerged immortalized epithelial cells overexpressing F508del CFTR (CFBE). Liessi et al. (2020) showed recently that CFTR targeted therapeutics, including an elexacaftor/tezacaftor/ivacaftor, changes ceramide and lysolipid pools.

The slow and incomplete correction of lipids by CFTR correctors in CF HBEC-ALI seems contrary to the general assumption that rescuing 10–20% of normal CFTR activity measured as forskolin-stimulated short circuit current would be sufficient to alleviate clinical symptoms, including downstream effects on inflammation and tissue remodeling.

Although we cannot simply extrapolate our *in vitro* observations to the *in vivo* situation, the inability to correct downstream abnormalities may help explain the variable and incomplete therapeutic effect of ivacaftor/lumacaftor in patients homozygous for F508del CFTR. Future studies using advanced 3D culture models of CF lung pathology, and in early CF patients, should establish whether elexacaftor/tezacaftor/ivacaftor, which shows 5–10 fold higher responses compared to ivacaftor/lumacaftor in some patients (Table 1), is also more effective in long term reduction of oxidative stress, lipid imbalance, basal and induced inflammation, and tissue remodeling in those patients who do not respond well to ivacaftor/lumacaftor.

### Persistent Enhanced Basal Cytokine Shedding in CF HBEC-ALI

Abnormal lipid metabolism in CF HBEC correlated with enhanced shedding of cytokines and growth factors to the basal medium (Table 2, Supplementary Table S1). The data highlight the enhanced inducible cytokine CXCL5 involved in neutrophil recruitment, tissue remodeling and COPD (Chen et al., 2019), and placental growth factor (PGF) which is induced in the lung hyperoxia response, involved in macrophage polarization and angiogenesis, and exacerbates pulmonary fibrosis (Zhao et al., 2019; Zhang et al., 2020). Both proteins correlated with oxidative stress markers, inflammation, and lung CT scores in CF infant BALF (Horati et al., 2020), consistent with a role of these epithelial factors in early CF lung disease. Interestingly the T-cell activator CCL17 was reduced in CF HBEC media (Figure 8, Table 2), which may be related to the low T-cell count in CF infant BAL (Scholte et al., 2019) and abnormal T-cell maturation in advanced CF lung disease (Hayes et al., 2020).

The antioxidants DFO and GSH partially corrected oxidative stress and lipid metabolism but did not significantly correct basal CF HBEC cytokine and growth factor profile in the HBEC model. The lack of efficacy of antioxidants suggests either that the level of correction is insufficient, or that enhanced basal cytokine shedding in CF airway cells is independent of oxidative stress and abnormal lipid metabolism. Another unexpected finding was that the most effective CFTR modulators available, which are in clinical use, were also unable to reduce basal inflammatory mediator and growth factor release. Further studies are needed to establish if this is due to insufficient CFTR correction or to epigenetic changes in CF that require more time or different signals to reduce inflammation, as has been extensively documented for asthma and COPD. Distinguishing these possibilities could lead to another approach for correcting CF lung disease that aimed at modulating epigenetic programming (Brasier and Boldogh, 2019; Ebenezer et al., 2019; Alashkar Alhamwe et al., 2020).

In contrast to DFO, extracellular GSH did reduce a subset of pro-inflammatory factors by more than 10-fold, including the pro-inflammatory cytokines CCL20, CSF-1, and MCP-1 that in BALF from CF infants are correlated with oxidative stress and lung disease (Horati et al., 2020). However, not all proteins enhanced in CF HBEC-ALI (Table 1) were affected. The explanation for distinct effects could lie in the different modes of action of DFO and GSH. DFO affects intracellular ROS production by sequestering intracellular Fe stores whereas the antioxidant GSH, which is extracellular and relatively impermeant, will likely also affect extracellular redox potential and exposed redox-sensitive signaling receptors. We have shown previously that GSH supplementation reduced the enhanced growth factor (AREG) and pro-inflammatory cytokine (IL6R) shedding in CF HBEC-ALI, by oxidative inactivation of the EGFR/ADAM17 axis in CF HBEC-ALI (Stolarczyk et al., 2016, 2018). Together, this suggests that modulation of intracellular and extracellular oxidative stress may contribute towards normalization of inflammation and tissue remodeling. N-acetylcysteine reduces inflammation and fibrosis in a murine model of Duchenne muscular dystrophy (mdx-; Burns et al., 2019). However, treatment of CF lung disease patients with available anti-oxidants, including NAC and GSH has not been successful (Ciofu et al., 2020), likely due to the unfavorable pharmacodynamics of these compounds.

### Lipid Ratios Affect Membrane Mediated Signaling

Basal levels of the two major ω-3 PUFA DHA and EPA (data not shown), followed the same pattern of responses to interventions in CF response. DHA and EPA differentially modulate membrane fluidity, lipid oxidation, and signal transduction (Sherratt and Mason, 2018) and are precursors of resolvins, bioactive lipids that control the resolution of inflammation. A combination of EPA and DHA can reduce induced inflammation in mouse models of asthma (Fussbroich et al., 2020). Treatment of F508del CFTR mice with a diet enriched in ω-3 PUFA reduces lung disease (Portal et al., 2018), however, a recent Cochrane meta-analysis of five limited trials did not indicate that ω-3 PUFA dietary supplementation is beneficial for CF patients (Watson and Stackhouse, 2020).

The role of lipid rafts and ceramide platforms in inflammasome activity and downstream inflammation and profibrotic pathways has been highlighted by several authors (Grassme et al., 2014; Li et al., 2019). VLCC deficient CERS2KO mice show enhanced LPS induced septic shock and EGFR/ADAM17 activity (Ali et al., 2015), consistent with our observations. A profound change in the lipid content of CF airway cells as we have demonstrated here may affect inflammasome responses under both basal and stimulated conditions. The possible effects of LCC/VLCC and AA/DHA ratios on inflammasome activity under basal conditions and during a challenge by pathogens should therefore be the subject of future studies. Although well-differentiated primary cell cultures *in vitro* have enabled us to focus on cell-autonomous phenomena in the epithelium, this approach is not a true 3D model of the airways and does not include the submucosal glands, immune system, or underlying connective tissue and blood vessels that may influence epithelial behavior. To address these complexities and establish the kinetics of cellular adaptation to CF pathology and pharmaceutical intervention, future studies of CF lung pathology should include long-term treatment using an advanced 3D culture model of CF airway epithelium.

### Summary and Conclusions

We have shown here that CFTR deficiency causes a closely similar pattern of oxidative stress and abnormal lipid metabolism in CF mouse lung, differentiated CF pig bronchial epithelial cells, and human bronchial epithelial cell. The results, therefore, establish this abnormality is an inherent property of CFTR deficient airway epithelial cells. In view of the incomplete correction of lipid balance and enhanced CF HBEC-ALI cytokine shedding by available CFTR targeted compounds, additional anti-inflammatory therapy may be required to reduce the effects of established CF lung disease.

## Data Availability Statement

The transcriptome datasets presented in this study can be found in online repositories. The names of the repository/repositories and accession number(s) can be found at: https://www.ncbi.nlm.nih.gov/geo/query/acc.cgi?acc=GSE154802.

## Ethics Statement

The animal study was reviewed and approved by Independent Committee on Ethical Use of Experimental Animals, Rotterdam (DEC 138-11-09). Human studies at Erasmus MC Rotterdam, were approved by the Medical Ethical Committee of the Erasmus MC (METC 2012-512), at McGill University, Montreal, Canada according to a protocol approved by the Institutional Ethics Protocol Review Board of McGill University (#A08-M70-14B).

## Author Contributions

All experiments were designed by BS and MV. BS supervised the study. Manuscript was written by BS and edited by JH, DR, and JBS. Preparation for measurement of lipids, and markers of oxidative stress analyses were done by JBS, TO, and DR. NK and AB provided the CFTR KO newborn pig lungs. MV and MS cultured CFTR KO pig and human primary cells. MV performed all experiments, the mouse breeding and tissue isolation, and analyzed the data. JS prepared and managed samples for analysis. The electrophysiology was performed and analyzed by MV, JL, and JH. Normalization and statistical analyses of lipidomics data was done by JBS and BS. Transcriptome analysis was performed by RB and EO, analyzed by BS and RB. All authors participated in reviewing the final version of the manuscript.

### Conflict of Interest

The authors declare that the research was conducted in the absence of any commercial or financial relationships that could be construed as a potential conflict of interest.

## Figures and Tables

**Table:** 

Term	Definition
AA	Arachidonic acid, ω-6, 20:4(n-6)
BALF	Broncho-alveolar lavage fluid
DHA	Docosohexaenoic acid, ω-3 [22:6(n-3)]
EPA	Eicosapentaenoic acid, ω-3 [20:5 (n-3)]
GSH	Glutahione (reduced)
LCC	Long carbon side chain (C14-16) ceramides
MDA	Malondialdehyde [HOC(H)=CH-CHO]
VLCC	Very long carbon chain (C22-26) ceramides
