# Supplementary material for: CFTR Correctors and Antioxidants Partially Normalize Lipid Imbalance but not Abnormal Basal Inflammatory Cytokine Profile in CF Bronchial Epithelial Cells
Source: Front Physiol. 2021 Feb 4;12:619442. doi: 10.3389/fphys.2021.619442 (PMC7891400; doi:10.3389/fphys.2021.619442)
Supplement: Supplementary file 1 [file Data_Sheet_1.pdf]

Supplemental data.

	NPX (Log2)	
**Assay**	**AV CF**	**AV WT**
**CXCL5**	7,66	4,71
**Gal-1**	4,52	2,46
**PGF**	8,40	6,22
**HO-1**	3,70	1,59
**IL18**	3,15	1,05
**CXCL11**	1,53	0,44
**CAIX**	7,09	5,99
**ADA**	4,23	3,10
**CASP-8**	3,01	1,92
**CCL17**	0,97	2,79
**TNFRSF21**	4,18	5,21
MMP12	4,46	3,09
CXCL1	11,26	12,28
TRAIL	3,15	4,37
ANGPT1	1,55	3,35
IL8	12,94	12,66
CSF-1	4,61	4,78
CXCL10	4,69	4,52
VEGFA	9,18	9,10
CCL20	10,26	9,75
MCP-1	5,46	4,86

	NPX (Log2)	
**Assay**	**AV CF**	**AV WT**
TNFRSF9	1,64	1,38
IL7	0,88	1,88
CD40	4,64	4,87
LAP TGF-b1	3,02	2,44
IL6	4,06	3,56
EGF	1,47	1,39
FGF2	3,57	3,86
Gal-9	4,47	5,36
PDGF B	0,69	0,68
CX3CL1	2,10	4,13
MMP7	7,33	9,51
MIC-A/B	2,51	2,23
TNFRSF12A	3,20	3,78
CD70	0,56	0,47
CXCL13	3,13	4,45
IL-1 alpha	1,09	-0,90
MUC-16	1,35	1,76
PD-L1	1,31	0,73
GZMB	1,53	0,54
ADGRG1	0,56	0,52

Below LOD in HBEC-ALI basal media						
TIE2	CD8A	TNFSF14	MCP-2	IL10	ANGPT2	TNF
MCP-3	CD4	IL33	CCL4	CCL23	PTN	CD83
CD40-L	NOS3	TWEAK	IL15	CD5	CXCL12	TNFRSF4
CD244	IL2	PDCD1	CD27	CCL3	IFN-gamma	
CRTAM	VEGFR-2	FASLG	IL5	ARG1	LAMP3	
MCP-4	GZMH	CD28	HGF	NCR1	ICOSLG	
CXCL9	KIR3DL1	CCL19	GZMA	DCN	IL4	
PD-L2	LAG3	IL13	KLRD1	IL12	IL12RB1	

Table S1. Basal shedding of cytokines and growth factors by primary CF and non-CF epithelial cells. Fluidigm-based protein array analysis (Olink 96x96 IMMUNO/ONCOLOGY) was applied to basal medium 24 h after changing the medium (BEGM-EGF) (see methods). CF HBEC-ALI (5 patients, Table 1, 31 samples) was

compared to non-CF (WT) (4 donors, see methods, 17 samples). Data are expressed as Average NPX (= -Log2 expression relative to the internal marker control on the array), ordered by the statistical significance of the difference between CF and Non-CF samples. A selection of these data is presented in Table 2*,* **In Bold:** Significantly different Cf compared to non-CF, Multiple T-test on merged data, discovery set at Padj <0.01, Q<0.02. **Upper panels** list all markers of inflammation and tissue remodeling that are detected above the lowest level of detection (LOD) of the array and above the Medium background (BEGM minus EGF) signal in all samples. **Lower panel** lists the markers tested that are not detected above LOD or medium control (BEGM minus EGF).

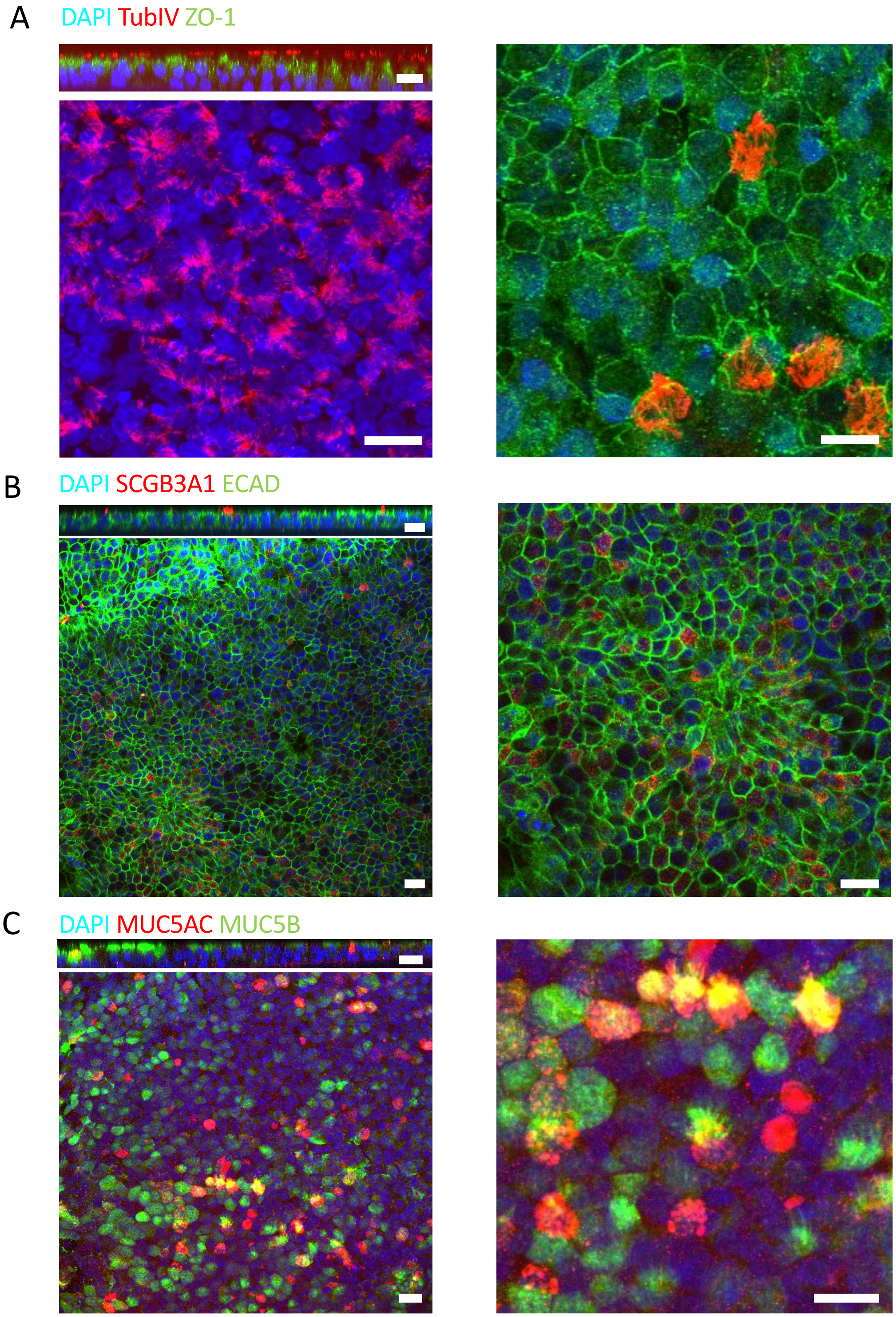
A
DAPI TubIV ZO-1
B
DAPI SCGB3A1 ECAD
C
DAPI MUC5AC MUC5B

D

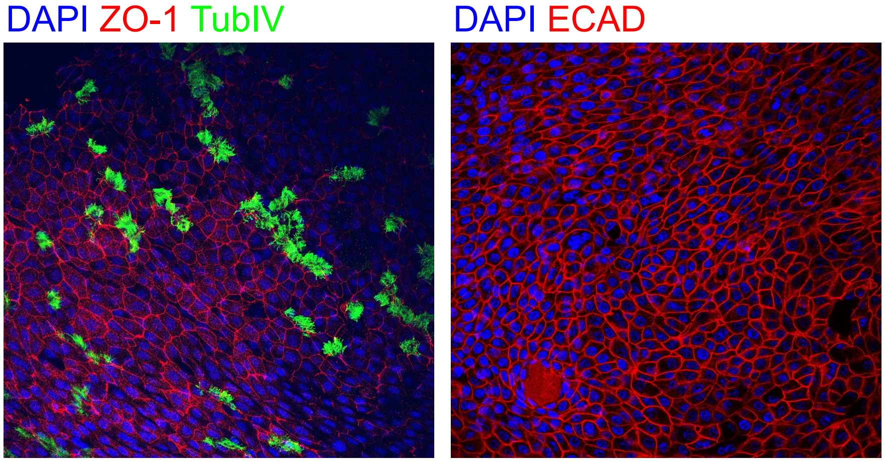

Figure S1. Immune fluorescence confocal analysis of primary airway cells. Human (**A-C**) and Pig (**D**) primary airway cells cultured on 12 mm transwell inserts with 0.4 µl pore polyester membrane (Costar) in air-liquid interface culture (BEGM, LONZA), as described in methods for 21 days. Filters used in experiments reported were visually inspected for leakage and ciliary activity during ALI culture. Each experimental group of donor filters was routinely checked for differentiation by staining a section for immune fluorescence microscopy as described in (62). DAPI (4′,6-diamidino-2-phenylindole, Thermo Fisher) was used as nuclear stain in all samples, white bars represent 20 μm. The selected panels show representative confocal images obtained with different antibodies and various donors, as indicated. **A, D: TubIV:** Beta tubulin IV, marker of cilia (apical), **ZO-1:** Tight junction protein-1, is detectable as a network in an apical focal plane, demonstrating the integrity of the apical-basolateral boundary. **B, D: ECAD:** E-Cadherin, epithelial cadherin (CDH1), marker of epithelial differentiation and lateral cell-cell interactions. **SCGB3A1:** Secretoglobin Family 3A Member 1, marker of epithelial secretory cell subsets, in particular Club (CLARA) cells in airways. Panels show dispersed secretory cells with apical secretory SCGB3A1 positive vesicles. **C: MUC5AC**, **MUC5B** mucins expressed in airway secretory cells, in particular goblet cells in airways.

Primary antibodies (anti human)

B-tubulin IV (species Mouse), BioGenex, dilution 1:100

ZO-1 (species Rabbit), Invitrogen, 1:100

Ecad (species Rabbit), Invitrogen 1:100

SCGB3A1 (species Mouse), R&D systems, 1:200

MUC5AC (species Mouse), Abcam, 1:500

MUC5B (Species Rabbit), Abcam, 1:500

Secondary antibodies

Alexa Fluor 488, 594 Donkey Anti rabbit, Jackson Immuno Research 1:500

Alexa Fluor 488, 594 Donkey anti mouse, Jackson Immuno Research 1:500

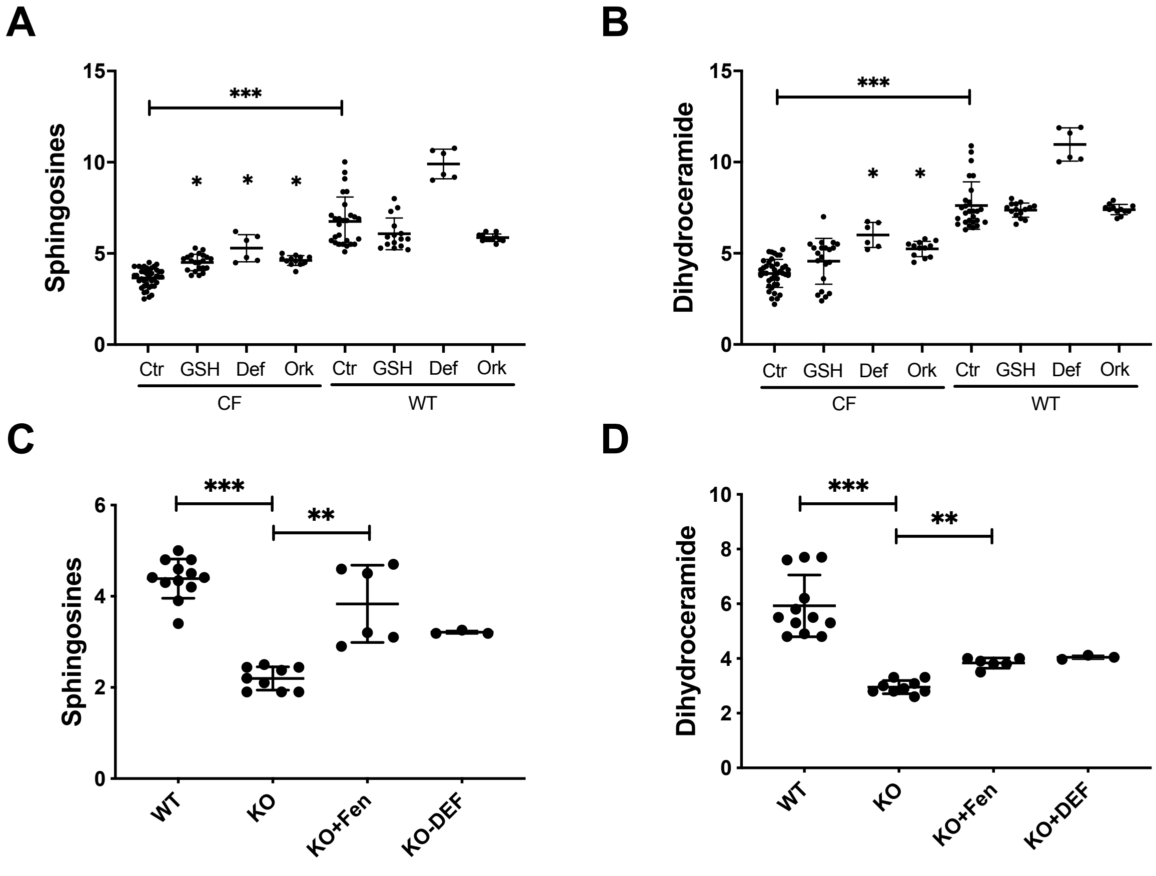

Fig S2 Abnormal Sphingosine and Dihydroceramide levels in Human and Pig CF BEC-ALI. Sphingosines and dihydroceramide expressed as pmol/nmol lipid phosphate. Datapoints refer to single inserts, averages +/- SEM are indicated. Samples, treatment, lipid collection and analysis as in Fig. 3, Fig. 5 and Fig 7 **(A,B)** CF and Non-CF HBEC-ALI: **Ctr:** carrier treated, merged data, CF: five donors (Table 1) (N=31), WT five donors (see methods) (N=24); **GSH**: treated with 10 mM glutathione, CF three donors (BCF000174, 554, 889; N=21), Non-CF: three donors (WP5,10,11; N=15); **Def** treated with deferoxamine, CF: two donors (BCF000191, 584, N=6), non-CF (two donors BD954, WP24, N=6). **Ork**: treatment with ivacaftor/lumacaftor (BCF000174, N=12, three independent experiment combined), parallel Non-CF controls (three donors, WP5,10,11; N=12). Merged HBEC ALI CF Ctr sphingosines and dihydroceramide differ from Non-CF (unpaired T-test, P<0.001, using averages of individual donors). GSH, Def and ivacaftor/lumacaftor have a significant but limited effect towards normal values

(unpaired t-test on combined data, comparing CF and parallel Non-CF). **(C,D) KO:** carrier treated PIG BEC-ALI CFTR KO (KO 3069, KO 3067, N=9). **WT**: CFTR WT littermate controls (3072, 3078, N=12), **KO+Fen** CFTR KO treated with fenretinide (KO 3069, KO 3067, N=6), KO+DEF, CFTR KO treated with deferoxamine (single donor, N=3). Sphingosine and Dihydrosphingosine is low in pig CFTR KO CF, as in human airway cells. Both fenretinide and deferoxamine had a modest but significant effect towards normal values (unpaired T-test compared to parallel controls).

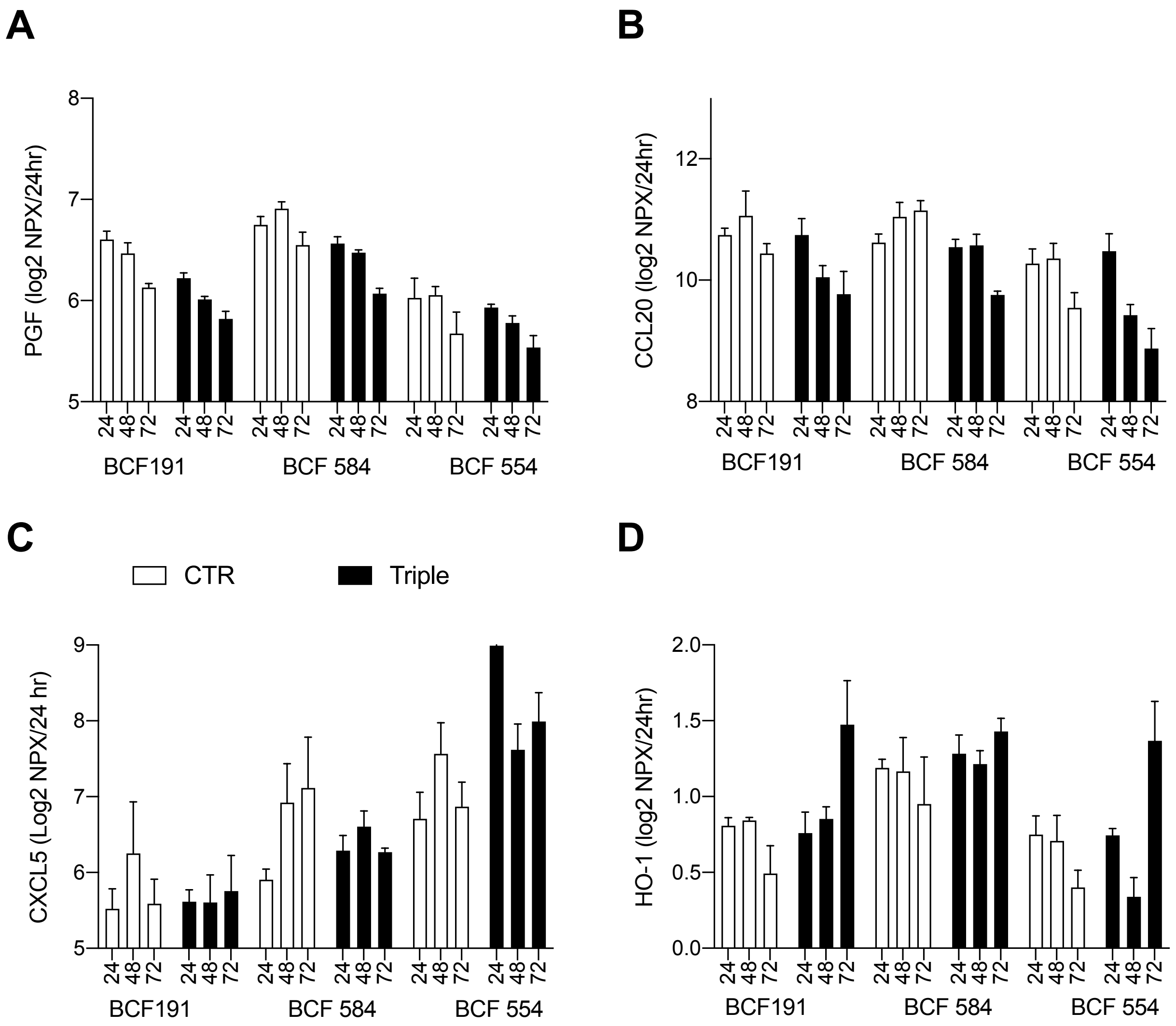

Fig. S3. Effect of triple therapy on cytokine and growth factor shedding.

Basal media obtained during the treatment of CF HBEC-ALI with ivacaftor/tezacaftor/elexacaftor (Figs. 7 and 8) were analyzed with the Olink INF/ONC array at 24, 48 and 72 hrs after treatment. Controls are parallel cultures treated with vehicle (Table 1, S2). Cells from three homozygous F508del CFTR patients showing medium (BCF000191), high (BCF000584), or low response (BCF000554) to ivacaftor/lumacaftor and ivacaftor/tezacaftor/elexacaftor (Table 1). Data represent expression relative to an internal control (normalized protein expression NPX, Log2 scale). **A**: PGF (placental growth factor) and **B**: CCL20 (pro-inflammatory cytokine) showed a downward trend with all donors, whereas for **C**: CXCL5 (pro-inflammatory chemokine) this was only observed with high responder BCF 584. In contrast the **D**:

HO-1 (heme-oxygenase) shedding appeared upregulated by Trikafta in CF cells. Other detectable cytokines (Table S1) did not change significantly (not shown).
